# Effusion detected by ultrasonography and overweight may predict the risk of knee osteoarthritis in females with early knee osteoarthritis: a retrospective analysis of Iwaki cohort data

**DOI:** 10.1186/s12891-022-05989-0

**Published:** 2022-11-28

**Authors:** Kyota Ishibashi, Eiji Sasaki, Daisuke Chiba, Tetsushi Oyama, Seiya Ota, Hikaru Ishibashi, Yuji Yamamoto, Eiichi Tsuda, Kaori Sawada, Songee Jung, Yasuyuki Ishibashi

**Affiliations:** 1 Department of Orthopedic Surgery, Hirosaki University Graduate School of Medicine, 5 Zaifu-cho, 036-8562 Hirosaki, Aomori Japan; 2 Department of Rehabilitation Medicine, Hirosaki University Graduate School of Medicine, Hirosaki, Aomori Japan; 3 Department of Social Medicine, Hirosaki University Graduate School of Medicine, Hirosaki, Aomori Japan; 4 Department of Digital Nutrition and Health Sciences, Graduate School of Medicine, Hirosaki University, Hirosaki, Aomori Japan

**Keywords:** Early knee osteoarthritis, Longitudinal analysis, Effusion, Overweight, Ultrasonography

## Abstract

**Background:**

Knee osteoarthritis (OA) has enormous medical and socioeconomic burdens, which early diagnosis and intervention can reduce. We investigated the influence of knee effusion on the progression of knee OA in patients with early knee OA.

**Methods:**

A total of 404 participants without radiographic knee OA were assessed from a 3-year longitudinal analysis. Participants were classified into non-OA and early knee OA groups. The effusion area (mm^2^) was quantified using ultrasonography. Receiver operating characteristic and logistic regression analyses were performed.

**Results:**

At the 3-year follow-up, 114 of 349 knees (32%) had progressed from non-OA and 32 of 55 knees (58%) had progressed from early knee OA to radiographic knee OA. Logistic regression analysis showed that female sex (odds ratio [OR] 3.36, 95% confidence interval [CIs] 2.98–5.42), early knee OA (OR 2.02, 95% CI 1.08–3.75), body mass index (OR 1.11, 95% CI 1.02–1.19), and effusion area (OR 1.01, 95% CI 1.01–1.02) were significantly correlated with knee OA progression. Women who were overweight (body mass index ≥ 25 kg/m^2^) with more severe effusion had a higher risk of OA progression (area under the curve = 0.691, OR = 6.00) compared to those not overweight (area under the curve = 0.568, OR = 1.91).

**Conclusion:**

Knee effusion may be an indicator of the progression of early-stage knee OA.

## Background

Knee osteoarthritis (OA) is one of the most common causes of chronic pain, stiffness, and disability, and has enormous medical and socioeconomic burdens [1–3]. OA was ranked as the 11th highest contributor to global disability in the Global Burden of Disease 2010 project [4]. Early diagnosis of knee OA and early intervention are important to reduce this increasing burden [5–8].

The prevalence of abnormalities detected on magnetic resonance imaging (MRI) and of bone marrow lesions, meniscal lesions, and synovitis were higher in patients with early knee osteoarthritis (EKOA) than in those with normal knees [9]. Among these abnormalities, synovitis is reportedly a trigger of cartilage degeneration and is associated with the symptoms and grades of knee OA [10, 11]. Although some cross-sectional studies have revealed that mild synovitis or effusion exist in symptomatic knees without radiographic knee OA [12, 13], to our knowledge, no longitudinal studies have yet investigated the influence of effusion on the progression of knee OA in patients with EKOA.

Knee synovitis is sometimes characterized by a palpable joint swelling, which can arise from synovial hypertrophy and effusion in the joint cavity [11]. Imaging evaluation of effusion includes MRI, ultrasonography, and arthroscopy; ultrasonography is a valid and highly reliable method of assessment of synovitis [14–16]. Our previous study established a method for the quantitative measurement of the suprapatellar effusion area using ultrasonographic images in patients with radiographic knee OA [17]. Suprapatellar effusion was also reported to be an imaging biomarker for predicting the development of knee pain [18].

## Methods

### Aim

This study aimed to compare normal and EKOA knees to investigate the influence of effusion on the progression of knee OA in participants without radiographic knee OA. It was hypothesized that greater effusion would be observed in patients with EKOA and that it would correlate with the progression of knee OA.

### Design

This was a retrospective follow-up study.

### Setting

Data from the Iwaki Health Promotion Project was used. This project is a community-based preventive program that aims to improve the average life expectancy by conducting general health check-ups, as described previously [19–22]. Since 2005, it has been conducted annually in the general population living in the Iwaki region of Hirosaki City (western Aomori prefecture, Japan).

### Participants

This study included participants who voluntarily participated in the Iwaki Health Promotion Project in 2016 and 2019. A total of 1,148 volunteers (459 men and 689 women) participated in the 2016 project. The participants answered questionnaires on their medical history, lifestyle, occupation, family history, health-related quality of life, and disease-specific information (such as knee symptoms). Here, we focused on participants without radiographic knee OA at baseline. We excluded 245, 282, 28, 4, and 3 participants whose radiographs revealed radiographic knee OA (Kellgren–Lawrence [KL] grade ≥ 2), whose ultrasonography data were incomplete, who did not undergo radiography, who had undergone knee arthroplasty, and who had a history of knee ligament injury or fracture, respectively. In total, 404 participants (182 men and 222 women) were followed up in the 2019 project and included in the analysis (follow-up rate: 68.9%). Participants with a body mass index (BMI) ≥ 25 kg/m^2^ were classified as overweight.

#### Radiographs

Knee radiographic examinations were performed using the CXDI-40EG Digital Radiography System. Experienced radiologic technicians and orthopedic surgeons obtained weight-bearing, full extension, and anterior–posterior radiographs of both knees with foot-map positioning upon check-up. The beam was positioned parallel to the floor at no angles and was aimed at the joint space. The sequencing was set at 60 kV, 50 mA, and 80 ms for all participants. Radiographic knee OA was diagnosed by a KL grade ≥ 2 in the more affected knee [23]. Only participants with no radiographic knee OA at baseline were included in the analysis. There were no participants with rheumatoid arthritis, ankylosing spondylitis, or psoriatic arthritis. At the 3-year follow-up, we defined knees with a KL grade of 0 or 1 as non-progressors and knees with radiographic knee OA as radiographic progressors. All joints were graded by two trained physicians (DC and ES) who had > 10 years of experience, and any discrepancies were resolved by mutual consultation.

### Suprapatellar effusion area evaluated by ultrasonography

Knee effusion was assessed based on the suprapatellar effusion area detected by ultrasonography, as described previously [17]. To reduce bias, all assessments of ultrasonography were performed by a trained physician (DC) with 10 years of experience in musculoskeletal ultrasonographic evaluation. The participants were examined in the supine position, with both knees semi-flexed and feet in the neutral position. The semi-flexed knee position was defined by placing a pillow beneath both popliteal areas. Longitudinal ultrasound scans of the suprapatellar region were obtained; a linear transducer (12 MHz, ViamoTM) was gently placed over the same area at the center and proximal poles of the patella. On these images, an echo-free space in the suprapatellar region was traced. The suprapatellar effusion area (mm^2^) was automatically calculated. In the present study, we did not evaluate the hypertrophic synovial soft tissue (i.e., we only evaluated the echo-free space). All ultrasound images were traced by a trained physician (SO). To investigate the intra- and inter-observer reliabilities of this measurement, 50 randomly selected ultrasonography images were traced and calculated by another orthopedic surgeon (HI) at a different time point. The intra- and inter-observer reliabilities were 0.99 (95% confidence interval [CI]: 0.987–0.996) and 0.94 (95% CI: 0.875–0.976), respectively.

### Classification criteria for early knee OA

EKOA was diagnosed according to the classification criteria proposed by the first international early knee OA workshop in Tokyo [8]. All participants answered the Knee Injury and Osteoarthritis Outcome Score (KOOS) questionnaire independently. The KOOS self-administered questionnaire consists of 42 knee-related items and five subscales: pain, symptoms, activities of daily living, sports and recreation (sports), and knee-related quality of life [24–26]. All items were scored from 0 to 4, and the scores were then summed. Next, the raw scores were converted to a 0–100 scale, with 100 representing the best result and 0 representing the worst. The classification criteria for EKOA were as follows: (A) KOOS: two of the four subscales (with the exception of KOOS sports) were required to score “positive” (i.e., ≤ 85%); (B) clinical examination: a minimum of joint-line tenderness or crepitus of the knee needed to be present; and (C) radiographs: KL grades of 0 or 1 at the standing, fixed flexion, and weight-bearing positions. Participants were divided into the non-OA group (i.e., without EKOA) and the EKOA group.

### Statistical analysis

Demographic data of the non-OA and EKOA groups are expressed as means ± standard deviations. Demographic data were compared between the two groups using the chi-squared test for categorical variables and the Wilcoxon rank-sum test for continuous variables, because some demographic parameters were not normally distributed according to the Shapiro–Wilk test. The effusion area was compared between the two groups using the Wilcoxon rank-sum test. Additionally, knees were divided into non-progressors and radiographic progressors and compared using an analysis of variance (ANOVA) and the Tukey’s post hoc test. To estimate the optimal cut-off effusion area for diagnosing EKOA and predicting the radiographic progressors, a receiver operating characteristic (ROC) analysis was performed. The effusion area values were used as a single variable in this analysis. A false-positive fraction was plotted against the true-positive fraction, and the cut-off value was defined by the Youden’s index (based on sensitivity and specificity), and the area under the curve (AUC) was calculated. Finally, multiple logistic regression analysis was conducted with the progression to knee OA at the 3-year follow-up as the dependent variable and with sex, BMI, prevalence of EKOA, and effusion area at baseline as the independent variables. Data input and analysis were performed using the JMP Pro software, version 13 (SAS Institute Inc., Cary, NC, USA). Statistical significance was set at *p* < 0.05.

## Results

**Fig. 1:**
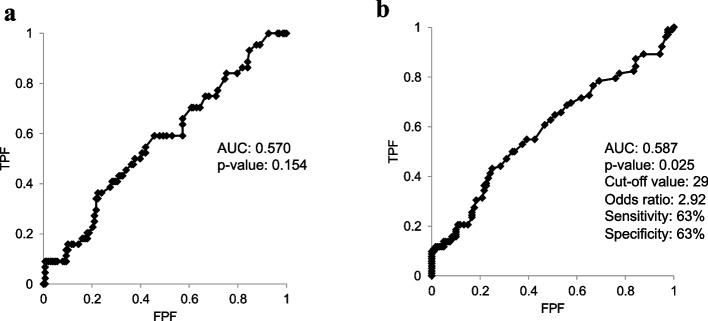
Receiver operating characteristic curves for the effusion area and early knee osteoarthritis diagnosis. **a** ROC curve for male participants and **b** ROC curve for female participants. ROC: receiver operating characteristic, TPF: true-positive fraction, FPF: false-positive fraction, AUC: area under the curve

Fifty-five of the 404 participants (14%) were classified into the EKOA group at baseline, with an EKOA prevalence of 10% in men and 21% in women. The overall mean age was 50.7 ± 13.3 years and the mean BMI was 22.7 ± 3.1 kg/m^2^ at baseline. The mean effusion areas detected by ultrasonography in the non-OA and EKOA groups were 39.9 ± 34.2 mm^2^ and 54.6 ± 39.5 mm^2^ in men (*p* = 0.048) and 31.2 ± 30.8 mm^2^ and 50.9 ± 48.1 mm^2^ in women (p = 0.002), respectively (Table 1). The number of patients with overweight was 88 (men; 50 and women; 36). ROC analysis revealed that the optimal effusion area cut-off value for diagnosing EKOA was 29 mm^2^ (AUC = 0.587, OR: 2.92, *p* = 0.025) in women (Fig. 1). The sensitivity and specificity were 63% and 63%, respectively. We were unable to determine the optimal cut-off value for effusion area in men (n.s.).

**Table 1:** Clinical characteristics of the participants

	Male			Female		
	Normal (***n***=165)	EKOA (***n***=17)	***p*** value	Normal (***n***=184)	EKOA (***n***=38)	***p*** value
**Age, years**	50.6±13.9	55.7±14.1	0.118	49.8±13.1	53.7±10.8	0.039
**BMI, kg/m** ^**2**^	23.5±2.8	24.6±2.3	0.092	21.7±2.8	23.4±4.6	0.027
**KOOS**						
** Symptom**	96.2±4.7	73.9±16.2	<0.001	95.5±5.1	78.5±14.2	<0.001
** Pain**	97.9±4.3	75.8±11.8	<0.001	97.8±4.0	75.4±14.2	<0.001
** Function**	99.5±1.3	87.9±17.4	<0.001	99.3±1.8	88.1±12.4	<0.001
** QOL**	94.5±9.1	55.9±17.4	<0.001	90.7±13.1	57.9±15.7	<0.001
**KLG 0, n (%)**	130 (78)	14 (82)	0.725	135 (73)	25 (66)	0.343
**Effusion- area (mm** ^**2**^ **)**	39.9±34.2	54.6±39.5	0.048	31.2±30.8	50.9±48.1	0.002

The values represent the demographic data of all participants, including participants with EKOA (EKOA group) and those without EKOA (non-OA group). Data are presented as mean±standard deviation for the age, BMI, KOOS, and effusion area. The KLG data are based on the number of participants (percentage of the whole population). The Wilcoxon rank-sum test was used to compare the mean values of the age, BMI, KOOS, and effusion area. The chi-squared test was used to compare the proportions of KLG between participants with and without OA.

*BMI* Body mass index, *EKOA* Early knee osteoarthritis, *KOOS* Knee injury and Osteoarthritis Outcome Score, *KLG* Kellgren–Lawrence grade, *OA* Osteoarthritis, *QOL* Quality of life

**Fig. 2:**
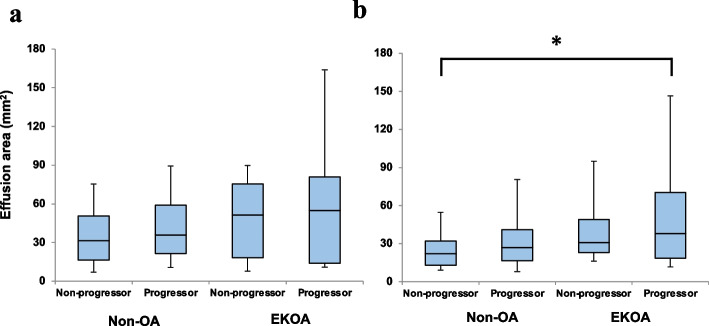
Effusion area at the baseline in non-progressors and radiographic progressors. **a** Box plots for male participants and **b** Box plots for female participants. Box plots show the median (central line inside the box), upper and lower quartiles (bounds of box), and the largest and smallest values. *p* < 0.05 indicates significance (*) in the ANOVA and the Tukey’s method. ANOVA: analysis of variance

**Fig. 3:**
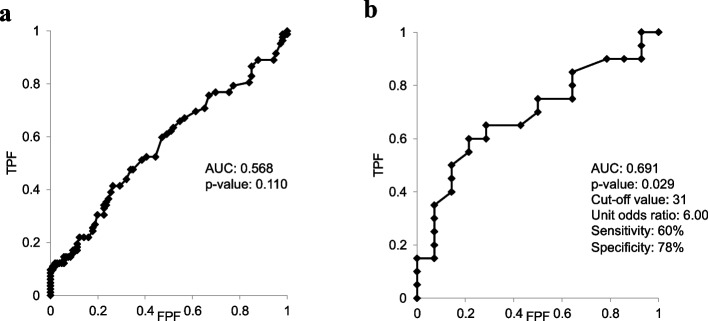
Receiver operating characteristic curves for the effusion area and for predicting the progression of knee osteoarthritis in participants who were and were not overweight. **a** ROC curve for male participants and **b** ROC curve for female participants. ROC: receiver operating characteristic, TPF: true-positive fraction, FPF: false-positive fraction, AUC: area under curve

At the 3-year follow-up, there were 37 (22%), 7 (41%), 77 (42%), and 25 (66%) radiographic progressors in the male non-OA, male EKOA, female non-OA, and female EKOA subgroups, respectively (Table 2). The proportion of radiographic progressors was significantly higher in the EKOA group than in the non-OA group (32 [58%] vs. 114 [32%], *p* = 0.003). Next, we compared the effusion areas between the non-progressors and radiographic progressors in the non-OA and EKOA groups. Radiographic progressors tended to show a greater effusion area, but a significant difference was only noted between the radiographic progressors in the female EKOA subgroup and non-progressors in the female non-OA subgroup (*p* = 0.006; Fig. 2). Logistic regression analysis revealed that female sex (OR 3.36, 95% CI 2.98–5.42), EKOA (OR 2.02, 95% CI 1.08–3.75), body mass index (BMI; OR 1.11, 95% CI 1.02–1.19), and effusion area (OR 1.01, 95% CI 1.01–1.02) were significantly correlated with knee OA progression (Table 3). Based on the results of the logistic regression analysis, ROC analysis was performed to determine the optimal cut-off value of the effusion area for predicting radiographic progressors in female participants with and without a BMI ≥ 25 kg/m^2^ (overweight). The cut-off value of the effusion area was 31 mm^2^ (AUC = 0.691, OR: 6.00, *p* = 0.029) in overweight women. The sensitivity and specificity were 60% and 78%, respectively (Fig. 3).

**Table 2:** Knee osteoarthritis in participants with non-osteoarthritis and early knee osteoarthritis at the baseline and changes over 3 years

	Male			Female			Total		
				Endpoint					
	Non-OA	EKOA	RKOA	Non-OA	EKOA	RKOA	Non-progressors	Radiographic progressors	*p* value
Baseline									
Non-OA	124 (75%)	4 (2%)	37 (23%)	100 (54%)	7 (4%)	77 (42%)	235 (68%)	114 (32%)	0.003
EKOA	7 (41%)	3 (18%)	7 (41%)	11 (29%)	2 (5%)	25 (66%)	23 (42%)	32 (58%)	

Values represent the number of participants. Numbers in parentheses indicate percentages. The chi-squared test was used to compare the proportion of patients with knee OA progression between participants with and without knee OA.

*EKOA* Early knee osteoarthritis, *OA* Osteoarthritis, *RKOA* Radiographic knee osteoarthritis

**Table 3:** Examination of factors related to the progression to knee osteoarthritis

	Univariate crude				Multivariate adjusted (stepwise)			
**Parameter**	β	*p*	OR	95% CI	β	*p*	OR	95% CI
**Female sex**	0.49	<0.001	2.67	1.73–4.12	0.61	<0.001	3.36	2.08–5.42
**BMI**	0.05	0.098	1.05	0.99–1.13	0.10	0.008	1.11	1.02–1.19
**Effusion area**	0.01	0.012	1.01	1.00–1.01	0.05	0.009	1.01	1.01–1.02
**EKOA**	0.52	<0.001	2.86	1.60–5.13	0.07	0.027	2.02	1.08–3.75

Multiple logistic regression analysis was performed with the progression to knee OA at 3 year follow-up as the dependent variable and with sex, BMI, prevalence of EKOA, and effusion area at the baseline as the independent variables, adjusted by age and Kellgren-Lawrence grade.

*p*<0.05 indicates statistical significance. β indicates adjusted correlation coefficient.

*BMI* Body mass index, *EKOA* Early knee osteoarthritis, *OR* Odds ratio, *CI* Confidence interval

## Discussion

This study has several important findings regarding risk factors of progression in patients with EKOA. The suprapatellar effusion area was higher in the EKOA group than in the non-OA group. Further, logistic regression analysis and ROC analysis revealed that a higher effusion area and higher BMI may be risk factors for knee OA progression. These results suggest that effusion may be associated with the progression of knee OA in the early stage.

OA is sometimes referred to as a non-inflammatory disease because the leukocyte count in the synovial fluid is lower in OA than in rheumatoid arthritis [27]. However, synovial activation and hypervascularization are positively correlated with the KL grade [17, 28–30]. Clinically, synovitis directly causes several knee symptoms, such as knee swelling, pain, and heat. Inflamed synoviocytes release several catabolic and proinflammatory mediators that lead to an overproduction of proteolytic enzymes, thereby causing cartilage degeneration [31]. Animal studies have assessed several treatments for synovitis that are designed to interfere with specific biological molecules, such as anti-tumor necrosis factor agents, anti-nerve growth factor antibodies, and antiproteases (anti-matrix metalloproteinases and anti-ADMATS) [32–34]. Some have reported that suppressing inflamed synoviocytes offers a protective effect on cartilage degeneration [11]. Thus, synovitis and effusion are important targets and biomarkers for knee OA. However, the influence and pathophysiology of knee effusion in EKOA remain unclear.

Knee effusion was found to be negatively correlated with the knee symptoms in both the early and late stages of knee OA [12, 13, 35]. Our previous MRI-based study investigated the prevalence of abnormalities on MRI, such as cartilage damage, bone marrow lesions, subchondral cysts, bone attrition, osteophytes, meniscal lesions, and synovitis, in general Japanese women without radiographic knee OA. Among these abnormalities, synovitis was positively associated with the presence of EKOA (odds ratio = 2.254, *p* = 0.002) and was most strongly associated with knee pain. Furthermore, we quantitatively evaluated the effusion volume on MRI. The effusion volume was higher in patients with EKOA than in those with normal knees [12]. In the present study, we evaluated the suprapatellar effusion area using ultrasonography, which showed the ability to evaluate synovial disease with an efficacy similar to that obtained with MRI and arthroscopy [14, 15]. Similar to our previous study, this study showed that the effusion area detected by ultrasonography was higher in patients with EKOA than in those with normal knees (even in male patients).

The KL grade is widely used to define radiographic knee OA [36]. However, knees with radiographic knee OA (KL grade ≥ 2) already exhibit irreversible structural changes. Luyten et al. proposed the following new EKOA classification criterion to intervene in knee OA before these irreversible changes occur [8]: no structural changes noted during standard radiographical examination. Although radiography is an attractive assessment method that can be used in clinical settings using standard tools, the pathogenesis and natural history of EKOA remain largely unknown. Recently, Mahmoudian et al. reported classification criteria that could contribute moderately to improve the detection of knees at a high risk of OA progression (sensitivity: 33% and specificity: 74%) from a longitudinal cohort study [37]. Our study also showed that the proportion of knees whose KL grades progressed over a 3-year follow-up was significantly higher in the EKOA group than in the non-OA group.

Obesity is a well-known risk factor for knee OA, and weight loss is recommended to reduce the symptoms of knee OA [38, 39]. The association between obesity and knee pain is multifactorial, involving a simple increase in the mechanical load on the joints through daily activity, as well as an increase in the systemic and local inflammatory load caused by cytokine production from the adipose tissue [40, 41]. A recent study reported that obesity was significantly associated with a greater prevalence and severity of synovial inflammation, as detected by MRI [42]. Sasaki et al. found that risk factors of EKOA were female sex, aging, obesity, and knee injury history [43]. Thus, obesity and synovitis are modifiable risk factors for knee OA at an early stage. Our data showed the presence of knee effusion seemed to play a crucial role in knee OA progression, particularly in women with obesity. When investigating synovitis, previous studies reported that weight loss significantly reduced the size of infrapatellar fat pad synovitis [44, 45]. Of course, overweight and obesity status should be discussed in the context of its modification through personal, clinical, and public strategy [46]. Additionally, our univariate crude analysis showed that only higher BMI was not associated with knee OA progression. This may be due to the relatively low prevalence of overweight and obese participants in this study (mean BMI = 23.3). The role of weight loss in the progression of early-stage knee OA is still unclear, but it may be a potential target for preventing knee effusion from worsening and the progression of knee OA.

This study has several limitations. First, it was a single-center study; thus, selection bias cannot be ruled out. Therefore, multi-center studies are needed to validate our findings. Second, the study participants were determined via voluntary participation of the community residents. Because this check-up was performed as part of an annual general health check-up, we could not control for the follow-up rate. In addition, the number of participants with incomplete ultrasonography data was high. However, the differences between included and excluded participants showed no significant differences. Third, effusion was only examined in the suprapatellar region. It remains unclear whether other quantitative measurements, such as thickening of the synovial membrane and other sites with knee effusion, are associated with knee synovitis. Additionally, this was an observational study, so no conclusions can be drawn regarding cause-effect for effusion or thickening of synovial membrane and symptoms. Fourth, the number of participants with EKOA were limited, particularly male participants. We could not determine the optimal cut-off value of the effusion area for diagnosing EKOA in men. Thus, conclusions regarding differences between men and women could not be drawn. Fifth, we did not perform US analysis in our 2019 study. Evolution of effusion area and BMI may be more valuable factors in terms of progression of knee OA. However, we were unable to find the significant associations between delta values of BMI (difference of BMI between baseline and the 3-year follow-up) and progression of knee OA (*p* = 0.87). Sixth, we did not evaluate participants in the short term (i.e., 1 and 2 years). Typically, knee OA is a disorder that slowly progresses. Our previous study reported that mild effusion synovitis presented in participants with EKOA [12]. As we thought that mild synovitis slowly progresses to knee OA, we decided to evaluate the participants at 3 years and for as long as possible. We intend to perform a short-term follow-up in future studies. Finally, the differences between the symptoms of normal aging and EKOA remain unclear [47, 48]. In addition, participants with EKOA at baseline had higher BMI and age. These factors may affect the knee effusion area. The pathophysiology of effusion in patients with EKOA should be assessed carefully.

Despite these limitations, our study has several significant findings that have important clinical relevance for intervention in the early stages of knee OA. We showed a relationship between the baseline effusion area and the progression of knee OA in female participants without radiographic knee OA. Our findings suggest that knee effusion may be involved in the pathophysiology of EKOA and may be used as an indicator of progressive early-stage knee arthritis.

## Conclusion

This study was the first to report on the influence of knee effusion on the progression of knee OA in patients with EKOA. Greater effusion area and an overweight status were identified as risk factors for the progression of knee OA, even in patients without radiographic knee OA. As this was an observational study, no conclusions can be drawn regarding causation between effusion and knee OA progression. However, effusion is one of the crucial pathologies in EKOA, and future studies should examine the cause of effusion in patients with EKOA.

## Data Availability

The datasets used and analysed during the current study are available from the corresponding author on reasonable request.

## Abbreviations

AUC: Area under the curve
ANOVA: Analysis of variance
BMI: Body mass index
CI: Confidence interval
EKOA: Early knee osteoarthritis
KOOS: Knee injury and Osteoarthritis Outcome Score
KL: Kellgren–Lawrence
MRI: Magnetic resonance imaging
OA: Osteoarthritis
OR: Odds ratio
RKOA: Radiographic knee osteoarthritis
ROC: Receiver operating characteristic
